# Immunogenicity of chimeric hemagglutinins delivered by an orf virus vector platform against swine influenza virus

**DOI:** 10.3389/fimmu.2024.1322879

**Published:** 2024-02-28

**Authors:** Gabriela Mansano do Nascimento, Pablo Sebastian Britto de Oliveira, Salman Latif Butt, Diego G. Diel

**Affiliations:** ^1^ Department of Population Medicine and Diagnostic Sciences, College of Veterinary Medicine, Cornell University, Ithaca, NY, United States; ^2^ Programa de Pós-graduação em Medicina Veterinária, Universidade Federal de Santa Maria, Santa Maria, Rio Grande do Sul, Brazil

**Keywords:** swine influenza, vaccines, chimeric HA, cross-protection, ORFV vectors

## Abstract

Orf virus (ORFV) is a large DNA virus that can harbor and efficiently deliver viral antigens in swine. Here we used ORFV as a vector platform to deliver chimeric hemagglutinins (HA) of Influenza A virus of swine (IAV-S). Vaccine development against IAV-S faces limitations posed by strain-specific immunity and the antigenic diversity of the IAV-S strains circulating in the field. A promising alternative aiming at re-directing immune responses on conserved epitopes of the stalk segment of the hemagglutinin (HA2) has recently emerged. Sequential immunization with chimeric HAs comprising the same stalk but distinct exotic head domains can potentially induce cross-reactive immune responses against conserved epitopes of the HA2 while breaking the immunodominance of the head domain (HA1). Here, we generated two recombinant ORFVs expressing chimeric HAs encoding the stalk region of a contemporary H1N1 IAV-S strain and exotic heads derived from either H6 or H8 subtypes, ORFV^Δ121^cH6/1 and ORFV^Δ121^cH8/1, respectively. The resulting recombinant viruses were able to express the heterologous protein *in vitro*. Further, the immunogenicity and cross-protection of these vaccine candidates were assessed in swine after sequential intramuscular immunization with OV-cH6/1 and OV-cH8/1, and subsequent challenge with divergent IAV-S strains. Humoral responses showed that vaccinated piglets presented increasing IgG responses in sera. Additionally, cross-reactive IgG and IgA antibody responses elicited by immunization were detected in sera and bronchoalveolar lavage (BAL), respectively, by ELISA against different viral clades and a diverse range of contemporary H1N1 IAV-S strains, indicating induction of humoral and mucosal immunity in vaccinated animals. Importantly, viral shedding was reduced in nasal swabs from vaccinated piglets after intranasal challenge with either Oh07 (gamma clade) or Ca09 (npdm clade) IAV-S strains. ﻿These results demonstrated the efficiency of ORFV-based vectors in delivering chimeric IAV-S HA-based vaccine candidates and underline the potential use of chimeric-HAs for prevention and control of influenza in swine.

## 1 Introduction

Orf virus (ORFV), a member of the *Parapoxvirus* genus within the *Poxviridae* family, holds great potential as a vector delivery platform for use in animals (1–6). The ORFV genome consists of a 138-kb double-stranded DNA molecule with 131 putative open reading frames (ORFs), some of which encode immunomodulatory proteins (IMPs). These IMPs play a role in modulating the host innate and proinflammatory responses to infection, although they are not essential for viral replication *in vitro* (7–10). These inherent immunomodulatory properties of ORFV-based vectors highlight their potential for use as vaccine delivery platforms with the ability to elicit protective immune responses against heterologous viral agents in livestock.

The advantages of using ORFV as a viral vector in animals include the restricted host range typically limited to sheep and goats, the self-limiting and non-systemic nature of the infection, and the lack of neutralizing antibodies against the vector, which is favorable for vaccine boosters (11–14). ORFV has been proven to induce long-lasting humoral and T-cell responses against heterologous antigens in multiple animal species (2, 12, 15). The ORFV strain D1701 has been shown to induce protective immunity against Pseudorabies virus (PRV) infection in pigs and cattle when used as a vector to express PRV glycoproteins (16). These findings complement two other independent studies testing heterologous prime–boost immunization with an ORFV recombinant expressing gC and/or gD of PRV (17, 18). ORFV vectors based on the D1701 or the IA82 strains were also used to express the Rabies virus (RABV) glycoprotein and elicited robust neutralizing responses against RABV in dogs, cats, pigs, and cattle (19, 20). In addition, a recombinant ORFV expressing the classical swine fever virus (CSFV) envelope glycoprotein E2 has also been shown to protect swine from CSFV challenge (2). ORFV recombinants expressing the porcine epidemic diarrhea virus (PEDV) spike (S) protein (OV-PEDV-S) elicited immunity in vaccinated piglets and passive immunity in piglets born to pregnant gilts immunized with the recombinant OV-PEDV-S (4, 6). Additionally, an ORFV recombinant expressing the HA protein of highly pathogenic avian influenza virus (HPAIV) H5N1 provided protection against lethal challenge and induced cross-clade (H5N1 clades 1, 2.2.2, and 2.2.3) and heterosubtypic (H1N1) immunity in mouse models (21). More recently, our team demonstrated that an ORFV vector expressing the full-length HA glycoprotein of a well-characterized swine influenza virus (OH07) elicited antibody and T-cell responses that correlated with protection against homologous IAV-S challenge (5). We have also shown that an ORFV vector expressing a centralized consensus H1 sequence elicited cross-reactive and protective responses against IAV-S challenge in pigs (22). Collectively, these studies validate ORFV as a valuable tool for the development of vaccines targeting various viral pathogens in swine and other animal species.

Swine influenza virus is a major cause of acute respiratory diseases in swine, with three main IAV-S subtypes H1N1, H1N2, and H3N2 currently circulating in swine (23). Beyond the economic burden that these outbreaks pose to the pork industry, there is a significant concern regarding the potential impact of these viruses on human health, since IAV-S has zoonotic potential, as evidenced by the 2009 influenza pandemic that was caused by a swine-origin H1N1 virus (24, 25).

The eight negative single-stranded RNA segments of IAV-S are within a lipidic virion envelope covered with surface HA and NA glycoproteins (26, 27). Predominantly, the virion is mostly surrounded by HA glycoproteins, which are the most immunogenic viral glycoprotein and play a crucial role in determining the host specificity, antigenicity, and pathogenicity of influenza A viruses (28, 29). The HA spikes are cylinder structures composed of a trimer of identical subunits (30). These subunits are formed by two disulfide-linked polypeptides resulting from proteolytic cleavage of the precursor HA0 protein into the membrane-distal HA1 head domain and the smaller, membrane-proximal HA2 stalk subunit (30, 31). The cleavage of HA0 is essential for the activation of membrane fusion and consequently, virus infectivity. The cleavage site consists of a single arginine residue for IAV-S HAs (32, 33). During virus infection, the HA has two main functions: receptor binding and membrane fusion (34, 35). HAs bind to the cell surface through sialic acid receptors and the virus can be taken into cells by endocytosis. The low pH in endosomes leads to conformational changes in HA1-HA2, which, in turn, result in HA2-mediated fusion of virus- and endosomal membranes (30, 35).

**Figure 1:**
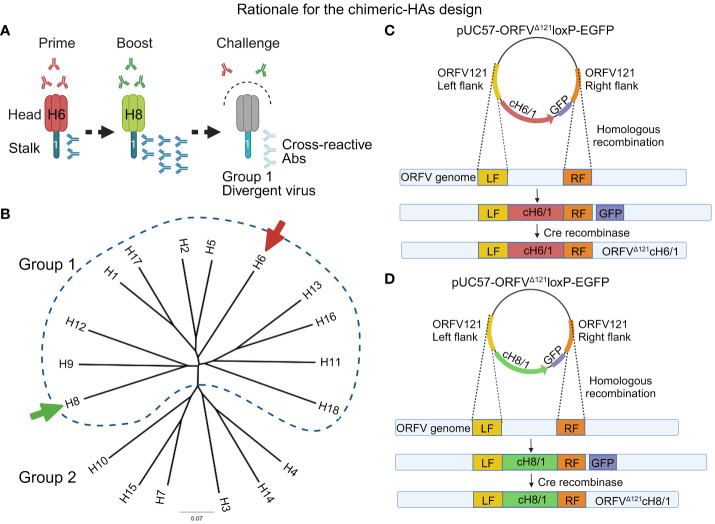
Overview of the chimeric vaccine strategy. **(A)** Sequential immunization with the chimeric HA constructs composed of exotic heads and sharing the same stalk domain belonging to the H1 subtype. The rationale for this approach is breaking the immunodominance of HA head by inducing a recall response against the conserved stalk, which may generate cross-reactive antibodies capable of protecting swine against divergent IAV-S. **(B)** HA-based phylogenetic tree representing the subclassification of influenza A virus subtypes into two groups (group 1 and 2). The arrows indicate the subtypes from group 1 used to design the head of the chimeric HAs containing a contemporary H1 stalk domain. **(C)** Representation of the construction of the recombinant plasmid for the generation of ORFV^Δ121^cH6/1 recombinant virus through homologous recombination. **(D)** Representation of the construction of the recombinant plasmid for the generation of ORFV^Δ121^cH8/1 recombinant virus through homologous recombination.

A comprehensive evolutionary study has provided evidence that the stalk domain of HA from different influenza subtypes has been evolving at a much slower rate than the head region, indicating the resilience of stalk cross-reactive epitopes to immune pressures (36). In order to enhance and broaden protection against influenza in pigs, novel vaccine platforms exploring the cross-reactivity of conserved domains have been developed. A promising approach focuses on inducing immune responses directed to the conserved HA2 domain of HA by generating chimeric HAs, which combine a common stalk domain, containing heads from distinct influenza subtypes that do not circulate in the target species. These chimeric HAs with exotic heads, when used in a heterologous prime–booster immunization regimen, redirect the immune responses towards the conserved and subdominant stalk domain thus breaking the immunodominance of the head domain (36–39). The expected outcome is that after the booster, a more robust immune response will be induced against the stalk, which displays higher cross-reactive potential against divergent IAV-S strains (**Figure 1A**). Recent studies revealed that sequential vaccination with chimeric HA constructs induced cross-reactive antibodies and protected mice and ferrets against divergent influenza strains (39–42). Additionally, a placebo-controlled phase I trial demonstrated that chimeric HA-based vaccines induce a strong long-lasting humoral response in healthy humans (43).

Here, we evaluated the efficacy of chimeric HAs delivered by the ORFV vector and assessed their ability to induce broad cross-reactive antibodies and protection against divergent influenza virus in swine.

## 2 Materials and methods

### 2.1 Cells and viruses

Primary ovine fetal turbinate (OFTu), swine turbinate (STu), and Madin–Darby Canine Kidney (MDCK) cells were used for viral propagation and cell-based assays. The cells were cultured in minimum essential medium (MEM), supplemented with 10% fetal bovine serum (FBS, heat inactivated), 2 mM of L-glutamine, and antibiotics (penicillin, streptomycin, and gentamicin). The wild-type orf virus (ORFV, OV strain IA82) was provided by Dr. Daniel Rock of the University of Illinois at Urbana-Champaign (7, 44, 45) and served as the parental virus to generate recombinant ORFV viruses that express heterologous HA proteins derived from the avian influenza virus (AIV, heads) and swine influenza virus (IAV-S, stalk). Both wild-type (wt) and recombinant OVs were propagated in primary OFTu cells.

Two H1N1 IAV-S strains, A/Swine/OH/24366/2007 (Oh07), from the gamma clade, and A/California/04/2009 (Ca09), from the “new pandemic” (npdm) clade, provided by Dr. Aradhya Gourapura of The Ohio State University were used to evaluate the efficacy of the recombinant vaccine candidates. Amplification of IAV-S was performed in MDCK cells using DMEM supplemented with HEPES buffer and TPCK-treated trypsin at a concentration of 2 µg/mL. Subsequently, these viruses were utilized for the virus challenge and antigen preparation for the whole-virus ELISAs.

For the whole-virus IgG ELISA, a panel of 10 additional IAV-S isolates (**Table 1**), representing all H1 circulating clades, was acquired from the National Veterinary Services Laboratory (NVSL, Ames, IA, USA). These viruses were propagated in MDCK cells as described above, purified through a sucrose cushion, and used as antigens on the ELISAs.

**Table 1:** Panel of the 12 IAV-S belonging to the H1 subtype used in different assays throughout this study.

Summary	Ohio/07	California/09	Iowa/20	Iowa/20	Michigan/20	Minnesota/17	Missouri/20	Oklahoma/17	Oklahoma/20	South Dakota/18	South Dakota/20	Texas/20
Year	2007	2009	2020	2020	2020	2017	2020	2017	2020	2018	2020	2020
Clade	Gamma	npdm	gamma	delta 1	npdm	alpha	npdm	delta 1	beta	gamma-2-beta-like	gamma	beta
State	Ohio	California	Iowa	Iowa	Michigan	Minnesota	Missouri	Oklahoma	Oklahoma	South Dakota	South Dakota	Texas
Isolate number	24366	4	A02524852	A02479151	A02524810	A01785306	A02479312	A02214419	A02245707	A02156993	A02524887	A02245632

### 2.2 Construction of recombination cassettes

To generate the two recombinant ORFV-SIV viruses evaluated in this study, chimeric HA (cHA) coding sequences were designed *in silico* and chemically synthesized and cloned into the pUC57 plasmid (GenScript^®^, Piscataway, NJ). Briefly, the cHAs were designed based on the stalk domain (nucleotide residues 1,033 to 1,698) of a contemporary H1N1 IAV-S strain [(A/swine/Minnesota/A02245569/2020 (H1N1) GenBank accession no. MT372532] and exotic head domains were selected to avoid IAV subtypes found in swine (H1 and H3) and those associated with HPAI (H5 and H7). The head domains (nucleotides 1 to 1,032) of the cHAs used in this study were based on avian influenza A viruses (AIV) belonging to either the H6 subtype [(A/American Green-winged Teal/Ohio/18OS2656/2018(H6N1) GenBank accession no. MN430905.1] or the H8 subtype [(A/Mallard/Ohio/18OS1248/2018(H8N4) GenBank accession no. MN431074.1] (**Figure 1B**) (cHA sequences are provided in the **Supplementary Material**). For the design of the chimeric HAs, cH6/1 and cH8/1, restriction endonuclease sites required for DNA insertion into the *ORFV121* locus of the ORFV genome were added, whereas HindIII and SalI restriction sites were added to the N- and C-terminus of cH6/1, and SpeI and SalI restriction sites were added to the N- and C-terminus of cH8/1. The Flag-tag epitope was added to the 3´ end, and either the p116 (46), an early/late ORFV internal promoter, or the vaccinia virus (VACV) late I1L promoter (47) were added to the 5´ end of the coding sequence of cH6/1 and cH8/1, respectively. Finally, potential poxviral transcription termination nucleotide sequences, (TTTTTNT) were removed from the chimeric HA coding sequence through silent nucleotide substitutions. The resulting synthetic DNA fragments were subcloned into the poxviral transfer vector pUC57-ORFV^Δ121^loxP-EGFP, resulting in two recombination cassettes, pUC57-ORFV^Δ121^cH6/1-loxP-EGFP and pUC57-ORFV^Δ121^cH8/1-loxP-EGFP. Correct cloning was confirmed by restriction enzyme, followed by indirect immunofluorescence assay (IFA) of transfected cells using a Flag-tag mouse monoclonal antibody (Thermo Fisher Scientific, Waltham, MA).

### 2.3 Generation of recombinant ORFV^Δ121^cH6/1 and ORFV^Δ121^cH8/1

To generate the recombinant ORFV^Δ121^cH6/1 and ORFV^Δ121^cH8/1 viruses, the full-length sequences of cH6/1 and cH8/1 were inserted into the *ORFV121* locus (7, 48) through homologous recombination between the parental OV-IA82 and the recombination plasmids pUC57-ORFV^Δ121^cH6/1-loxP-EGFP or pUC57-ORFV^Δ121^cH8/1-loxP-EGFP (**Figures 1C, D**) using Lipofectamine 3000 (Life Technologies, Carlsbad, CA), as described by Joshi et al. (2021) (5). The presence of the chimeric HAs and the absence of the wild-type virus were confirmed by PCR amplification and electrophoresis analysis in 1% agarose gel using two pairs of primers designed for the stalk domain of H1, and an internal region of *ORFV121*, respectively. The primers for the stalk domain of the chimeric HAs were Fw-5´-ACTGCGGTACCTATTTAAAAGTTGTTTGGTGAACTTAAATGGG-CCTATTCGGGGCCATTGC-3´ and Rv-5´-GAGGTCTCGAGTTACTTGTCGTCATCGTCTTTG-TAGTCAATCT-GGTAGATCTTTGTTGAGTCCAGC-3´ (637 bp), while the primers for the *ORFV121* (401 bp) were previously described (49). PCR was performed using Q5^®^ Hot Start High-Fidelity DNA Polymerase (New England Biolabs, Ipswich, MA, USA), following the recommendations from the manufacturer. For each reaction, specific annealing temperatures were utilized based on the optimal melting temperature (Tm) of the primers used (62°C for amplifying the cHA1 stalk fragment and 56.5°C for amplifying a fragment of the ORF121 locus). To validate the presence and integrity of the heterologous genes and ensure the preservation of OV IA82 identity and integrity with the deletion of the *ORFV121*, whole genome sequencing was performed using the MinION *Mk1C* sequencing platform (Oxford Nanopore Technologies, ONT).

### 2.4 *In vitro* characterization of ORFV^Δ121^cH6/1 and ORFV^Δ121^cH8/1

Replication kinetics of ORFV^Δ121^cH6/1 or ORFV^Δ121^cH8/1 recombinant viruses were assessed through single- and multi-step growth curves in OFTu and STu cells and compared to the parental OV-IA82, as previously described (5). Briefly, for the multistep and single-step growth curves, the cells were infected at 0.1 MOI and 10 MOI, respectively, and subsequently harvested at 6, 12, 24, 48, and 72 hours post-infection (hpi). The controls were mock-infected OFTu and STu collected at 0 hpi. Viral titers were determined at each time point by limiting dilution and expressed as tissue culture infectious dose 50 (TCID_50_) per milliliter.

To evaluate the expression of heterologous genes by the recombinant viruses, OFTu cells were infected with either ORFV^Δ121^cH6/1 or ORFV^Δ121^cH8/1, and evaluated by indirect IFA, flow cytometry, and Western blot, as previously described (49). Anti-FLAG tag epitope monoclonal mouse antibody (Sigma-Aldrich) was used for the detection of the fusion cH6/1- or cH8/1-flag proteins in all assays.

The stability of both cH6/1 and cH8/1s genes inserted into the *ORFV121* locus was evaluated during 10 serial passages of the respective recombinant viruses in OFTu cells by assessing expression through IFA by using a monoclonal anti-FLAG M2 mouse antibody (1:500, F1804-5MG, Sigma-Aldrich), as previously described (49).

### 2.5 Immunization-challenge study in swine

The ability of ORFV^Δ121^cH6/1 or ORFV^Δ121^cH8/1 to induce immune responses and protection was evaluated in pigs. A total of 28 3-week-old weaned cross-bred piglets obtained from a high health-status herd (Midwest Research Swine, Glencoe, MN) were used in the study. The animals were seronegative for the subtypes H1N1, H1N2, and H3N2 by ELISA, and for influenza A virus as determined by real-time PCR testing targeting the Matrix gene. Animals were randomly allocated to four experimental groups as follows: group 1, ORFV^Δ121^cH6/1 (prime) + ORFV^Δ121^cH8/1-IAV-S (boost) immunized/Oh07 challenged (*n* = 7); group 2, ORFV^Δ121^cH6/1 (prime) + ORFV^Δ121^cH8/1-IAV-S (boost) immunized/Ca09 challenged (*n* = 7); group 3, sham-immunized/Oh07 challenged (*n* = 7); and group 4, sham-immunized/Ca09 challenged (*n* = 7) (**Supplementary Figure S1**).

After one week of acclimation, immunizations were performed (4 weeks of age) by intramuscular injection of either 2 mL of a virus suspension containing 10^7.38^ TCID_50_ mL^−1^ (groups 1 and 2) or 2 mL of MEM (groups 3 and 4). Animals were immunized on day 0 (D0) and boosted on day 21 (D21). Two divergent viruses were selected for the challenge: Sw/OH/24366/2007 (H1N1) (Oh07) (31) and A/California/04/2009 (H1N1) (Ca09). On day 35 (D35), pigs in groups 1 and 3 were challenged intranasally (using a needle-free 3-mL syringe) with 1 mL of a virus suspension containing 1×10^7^ TCID_50_ mL^−1^ of IAV-S Oh07, while groups 2 and 4 received an intranasal challenge with 1×10^7^ TCID_50_ mL^−1^ virus suspension of IAV-S CA09 (**Supplementary Figure S1**).

Throughout the study, animals were monitored daily for clinical signs of influenza infection. Serum and whole blood samples were collected on days 0, 21, 28, and 35 post-immunization (D0, D21, D28, and D35), as well as on days D38 [3 days post-challenge (dpc)], D40 (5 dpc), and D42 (7 dpc). Nasal swabs were collected at 0 dpc, 3 dpc, 5 dpc, and 7 dpc. On day 7 pc (D42), animals were subjected to euthanasia (captive bolt plus exsanguination) following the American Veterinary Medical Association (AVMA) guidelines for the euthanasia of animals. Prior to euthanasia, animals were tranquilized/sedated with acepromazine (0.44 mg/kg) and xylazine (0.2 mg/kg) given via intramuscular injection. The animal study was conducted at Cornell University, adhering to the guidelines and protocols approved by the Institutional Animal Care and Use Committee (IACUC approval no. 2019-0041) and in accordance with the Animal Welfare Act Amendments.

### 2.6 Antibody isotype ELISAs

Levels of specific IgG antibodies in sera and IgA antibodies in the bronchoalveolar lavage (BAL) were determined against 12 distinct whole-virus IAV-S (**Table 1**, **Supplementary Figure S2**) using a previously described in-house ELISA (49), with some modifications. This isotype-specific ELISA used sucrose-concentrated, whole IAV as antigen to coat the ELISA plates (Thermo Fisher Scientific, Immulon 1B catalog no. 3355), allowing detection of antibodies to a specific virus. For the IgG ELISA, serum was diluted at 1:50, while for the IgA ELISA, BAL was diluted at 1:20, added to the coated ELISA plates and incubated for 3.5 h at 37°C. The ELISA plates were coated with 500 ng/well of heat-inactivated sucrose cushioned whole viruses overnight at 4°C. Goat anti-pig biotinylated antibody IgG or IgA (Bethyl Laboratories, TX, USA; catalog no. A100-104 or A100-102A, respectively) was used at 1:2,000 in blocking buffer, composed of 5% non-fat dry milk in Phosphate-buffered saline (PBS) with 0.05% Tween 20 detergent (T-PBS).

The serum samples were evaluated by measuring their optical density (OD) at 450 nm using a microplate reader (SYNERGY LX, BioTek, Winooski, VT, USA). The OD values were used as the relative measure of antibody level (50). To standardize the OD values, each test and control sample was compared to the OD value of an uncoated well. Prior to the actual testing, all assay formats were optimized using serum samples from animals with known serological status. Each sample was tested two times independently and results presented here represent one replicate in which all experimental samples were tested side by side.

### 2.7 Viral load in nasal secretions and tissues

Viral load in nasal secretions, BAL, and lungs was determined by real-time reverse transcriptase polymerase chain reaction (rRT-PCR). Lung tissue lysates were prepared for RNA extraction using 2 g of lung tissue of individual pigs. Tissues were homogenized in 20 mL of MEM. Approximately 200 µL of the tissue homogenate supernatant was subjected to RNA extraction using the MagMax Core extraction kit (Thermo Fisher, Waltham, MA, USA) and the KingFisher flex automated extraction platform as previously described (49). Real-time RT-PCR using the Path-ID™ Multiplex One-Step RT-PCR kit (Thermo Fisher, Waltham, MA, USA) targeting the conserved matrix (M) gene (Integrated DNA Technologies, Coralville, IA, USA) was used to detect RNA from the influenza A virus. The probe and forward and reverse primers used were 5′-FAM-TCA GGC CCC CTC AAA GCC GA-BHQ1-3′, 5′-AGA TGA GTC TTC TAA CCG AGG TCG-3′, and 5′-TGC AAA AAC ATC TTC AAG TCT CTG-3′, respectively. The cycling conditions for amplification of the M gene target were 10 min at 48°C for reverse transcription, 10 min at 95°C for polymerase activation, 45 cycles of 15 s at 95°C for denaturation, and 1 min at 60°C for annealing and extension, which was performed using the CFX96 Touch Real-Time PCR Detection System (Bio-Rad, Hercules, CA, USA). Standard curves were established using 10-fold serial dilutions ranging from 10^−1^ to 10^−8^ of either Oh07 or Ca09 virus stocks. The relative genome copy number (copies mL^−1^) (log10) of each sample was derived from the CTs obtained for the established standard curves by CFX Maestro software (Bio-Rad), as previously described (49). Appropriate positive and negative controls were added to each extraction and PCR plates to be run along with test samples.

Additionally, infectious virus in nasal swabs and tissue samples was assessed in MDCK cells and expressed as TCID_50_ mL^−1^, as described in previous studies (5, 49). The mouse monoclonal antibody (mAb) used to assess IAV-S infectivity in the viral titration by IFA was provided by Drs. Eric Nelson and Steve Lawson of South Dakota State University (IAV-NP HB-65 462 mAb; at 1:500). This antibody targets the conserved nucleoprotein (NP) of influenza A viruses. The presence of fluorescent foci indicated virus-positive wells.

### 2.8 Statistical analysis

GraphPad Prism software v9.0 (GraphPad Software, San Diego, CA, USA) was used for all statistical analyses. Shapiro–Wilk test was performed to verify data normality, followed by either *t*-test or unpaired *t*-test to compare the means between the groups for either normal or non-normal data, respectively. Tukey’s multiple comparison post-test was performed for pairwise comparison. A *p*-value lower than 0.05 was considered significant. The flow cytometry data were acquired using the Attune NxT Flow Cytometer and analyzed with FlowJo V10 software (FloJo, Ashland, OR, USA).

## 3 Results

### 3.1 *In vitro* characterization, replication kinetics, and expression of the heterologous proteins by ORFV^Δ121^cH6/1 and ORFV^Δ121^cH8/1 viruses

**Figure 2:**
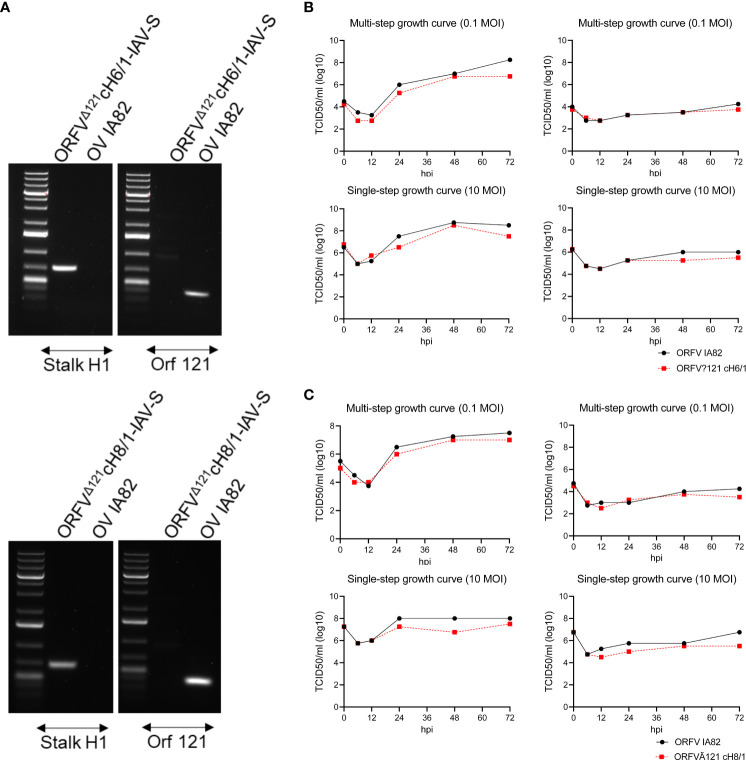
Characterization of ORFV^Δ121^cH6/1 and ORFV^Δ121^cH8/1. **(A)** Agarose gel demonstrating that an H1 stalk fragment was amplified, confirming its presence in both recombinant viruses, and the lack of amplification of ORF121 gene, indicating that the recombinant ORFV^Δ121^cH6/1 and ORFV^Δ121^cH8/1 viruses were purified. Growth curves comparing the replication of recombinant **(B)** ORFV^Δ121^cH6/1 and **(C)** ORFV^Δ121^cH8/1 viruses and the parental OV-IA82 virus at different time points in OFTu (left) and in STu (right) cells.

Following recombinant ORFV selection and purification, PCR amplification (**Figure 2A**) and whole genome sequencing confirmed the integrity of the inserted chimeric HA sequences in the ORFV^Δ121^cH6/1 and ORFV^Δ121^cH8/1 viruses (GeneBank accession no. PP211528 and PP211529; BioProject ID. PRJNA1068754). Replication kinetics of ORFV^Δ121^cH6/1 and ORFV^Δ121^cH8/1 viruses were assessed in OFTu and STu cells by multi- and single-step growth curves (**Figures 2B, C**). Both viruses replicated to high titers in OFTu cells, while a markedly impaired replication was observed in primary swine cells.

**Figure 3:**
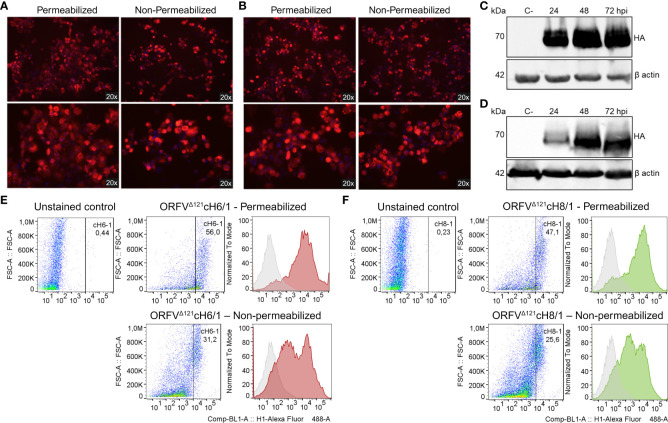
Expression of heterologous proteins by ORFV^Δ121^cH6/1 and ORFV^Δ121^cH8/1 recombinant viruses. Expression of chimeric HAs (red fluorescence) by the recombinant **(A)** ORFV^Δ121^cH6/1 and **(B)** ORFV^Δ121^cH8/1 viruses was assessed by IFA in permeabilized (left) and non-permeabilized cells (right) infected with 1 MOI (48 hpi). **(C)** Increasing levels of cH6/1 (~70 kDa) and **(D)** cH8/1 in ORFV^Δ121^cH6/1-infected and ORFV^Δ121^cH8/1-infected ovine cells, respectively, as detected by Western blot. Mock-infected OFTu cells were used as negative controls, while beta actin was used as loading control for the WB. **(E)** The flow cytometry data were consistent with the two previous assays, showing a slightly higher expression of cH6/1 than **(F)** cH8/1 at 72 hpi. OV-IA82-infected OFTu cells were used as negative controls for the gating strategy, where the gated cells indicate positive cell population.

The expression of cH6/1 and cH8/1 by the respective recombinant viruses was verified through IFA, WB, and flow cytometry using an anti-FLAG mAb. Chimeric protein expression in ORFV^Δ121^cH6/1-infected (**Figure 3A**) and ORFV^Δ121^cH8/1-infected (**Figure 3B**) cells was observed within the cytoplasm (permeabilized) and on the cell surface (non-permeabilized). Notably, the levels of the chimeric HA proteins (~70 kDa) increased up to 72 hpi in OFTu cells infected at 10 MOI by the recombinant ORFV^Δ121^cH6/1 (**Figure 3C**) and ORFV^Δ121^cH8/1 viruses (**Figure 3D**). The flow cytometry data confirmed the findings from IFA and WB assays (**Figures 3E, F**). The expression of cH6/1 and cH8/1 by the recombinant ORFV^Δ121^cH6/1 and ORFV^Δ121^cH8/1 viruses was observed in approximately 56% and 47% of the cells intracellularly and in 31% and 25% of the plasma membrane of infected cells, respectively.

### 3.2 Humoral immune response in swine

**Figure 4:**
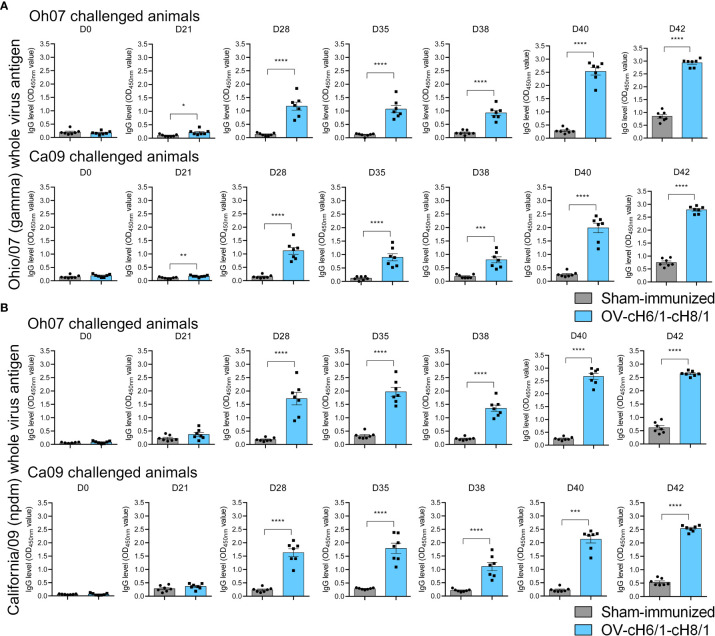
Dynamics of antibody responses to immunization. Increasing IgG responses induced by priming with ORFV^Δ121^cH6/1 and booster with ORFV^Δ121^cH8/1-IAV-S in Oh07 and Ca09 challenged pigs were assessed by whole-virus ELISA for the challenge virus **(A)** Oh07 and **(B)** Ca09 at the indicated time points post-immunization. *p*-values: *p < 0.05, **p < 0.01, ***p < 0.001, and ****p < 0.0001.

IAV-S-specific total IgG antibodies were detected as early as 3 weeks post prime-vaccination and increased markedly after the booster immunization on D21 continued to increase until D42 (7 dpc) in animals that received the sequential immunization with ORFV^Δ121^cH6/1 followed by the booster with ORFV^Δ121^cH8/1 against both challenge viruses (**Figure 4A**). Similar results were detected using either Oh07 or Ca09 purified viruses as the antigen in the ELISA (**Figure 4B**). An anamnestic response was observed in both vaccinated and sham-immunized groups following the experimental challenge infection. Notably, IgG levels reached their peak at D42 (or 7 dpc) and were significantly higher in vaccinated pigs, suggesting that the challenge infection boosted the vaccine-specific memory responses.

**Figure 5:**
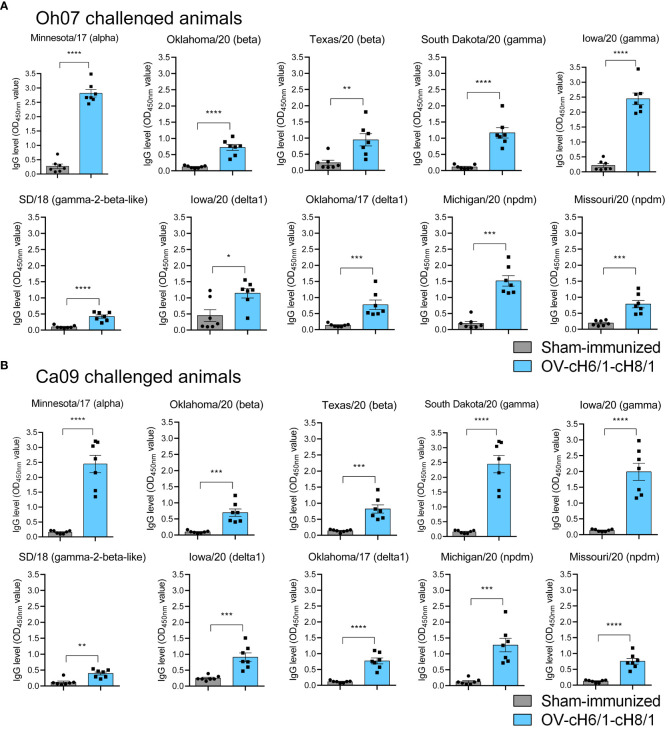
Humoral responses to immunization at day 35 was assessed by whole-virus ELISA against a panel of 10 divergent viruses. IgG antibody levels by prime with ORFV^Δ121^cH6/1 and booster with ORFV^Δ121^cH8/1-IAV-S in **(A)** Oh07 and **(B)** Ca09 challenged animals. *p-*values: *p < 0.05, **p < 0.01, ***p < 0.001, and ****p < 0.0001.

The breadth of IAV-S specific IgG responses induced by immunization was also assessed against a panel of 10 divergent viruses on D35 (0 dpc) (**Table 1**). The animals that received the prime and booster with the recombinant ORFV^Δ121^cH6/1 and ORFV^Δ121^cH8/1 viruses presented significantly higher IgG responses (**Figures 5A, B**). These results demonstrate a broad binding capability of the antibodies elicited by intramuscular (IM) immunization of the chimeric viruses in pigs.

### 3.3 Mucosal immunity in lungs

**Figure 6:**
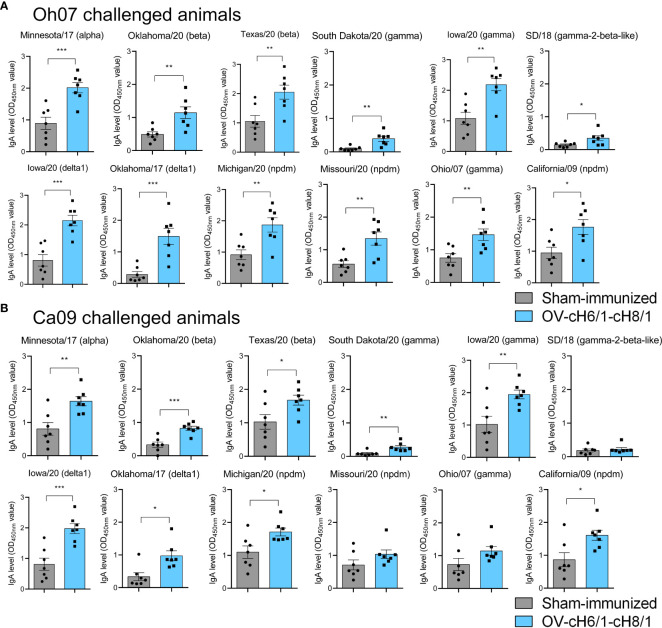
Mucosal antibody responses to immunization were assessed in BAL by whole-virus ELISA against a panel of 10 divergent viruses at D42. IgA antibody levels induced by prime with ORFV^Δ121^cH6/1 and booster with ORFV^Δ121^cH8/1 in **(A)** Oh07 and **(B)** Ca09 challenged animals is shown in blue. *p*-values: *p < 0.05, **p < 0.01, and ***p < 0.001.

The mucosal antibody responses in vaccinated compared to sham-immunized pigs were assessed by measuring IgA antibody levels in BAL post-euthanasia at D42 against the same panel of divergent viruses used in previous ELISAs. Immunized animals within the Oh07 challenge group presented significantly higher levels of IgA against all the 12 IAV-S viruses compared to the sham-immunized swine (**Figure 6A**). On the other hand, vaccinated swine challenged with the Ca09 virus showed significantly higher IgA responses for all divergent viruses except SD/18, Missouri/20, and Oh/07, which belong to gamma-2-beta-like, npdm, and gamma clades, respectively (**Figure 6B**). Although the challenge infection led to anamnestic antibody responses, the increased levels of IgA antibodies in vaccinated animals compared to the sham-immunized animals suggest that cross-reactive immune responses were elicited by IM immunization with the recombinant ORFV^Δ121^cH6/1 and ORFV^Δ121^cH8/1 viruses in swine.

### 3.4 Viral shedding in nasal secretions

**Figure 7:**
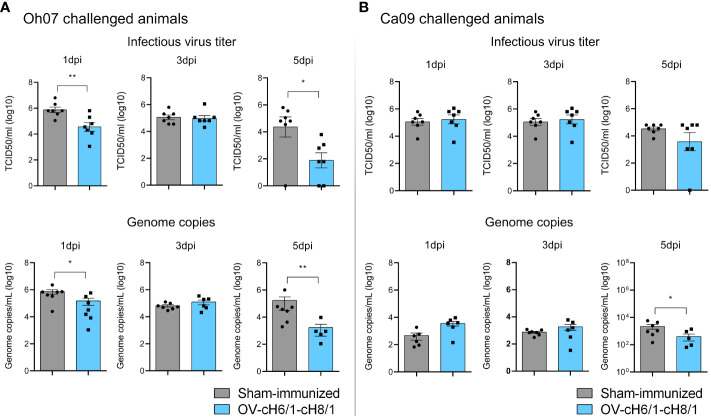
Shedding of IAV-S through nasal secretions. Viral shedding in **(A)** Oh07 challenged pigs and **(B)** Ca09 challenged pigs was determined by infectious titer (upper) and (lower) genome copy numbers. *p*-values: *p < 0.05 and **p < 0.01.

The presence of viral RNA and infectious virus in nasal secretions was assessed after an intranasal challenge with either Oh07 or Ca09 viruses. Animals immunized with OV-cH6/1-cH8/1 viruses and subsequently challenged with Oh07 showed a 10-fold decrease in infectious virus shedding through nasal secretions on 1 dpc, and this difference reached 20-fold at 5 dpc, compared to the respective sham-immunized group (**Figure 7A**). Similar results were observed in viral RNA load as determined by RT-qPCR. The peak virus shedding for the OV-cH6/1-cH8/1+Oh07 challenged pigs was 3 dpc, showing no significant increase in infectious virus and genome copy numbers from 1 dpc to 3 dpc. Taken together, these findings suggest an early control of Oh07 replication and early viral clearance in immunized animals. No significant differences were found in the Ca09 challenged groups on 1 and 3 dpc, but a slight decrease in the viral RNA was observed in the vaccinated group at 5 dpc (**Figure 7B**). For both challenge groups, no infectious virus or IAV-S genome copies were found at 7 dpc. These results demonstrated that vaccination with OV-cH6/1-cH8/1 led to a decrease in viral shedding in nasal secretions after Oh07 infection but not Ca09.

### 3.5 Lung lesions and viral load

**Figure 8:**
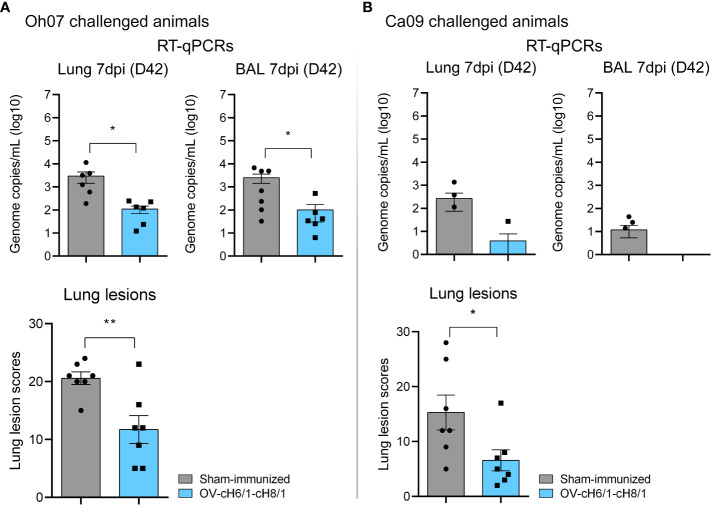
Lung scores and viral load in target tissues. **(A)** Lung lesions and presence of genomic RNA locally in swine challenged with Oh07. **(B)** Similarly, lung lesion scores and viral load were evaluated in Ca09 challenged animals. *p*-values: *p < 0.05 and **p < 0.01.

Macroscopic lesions seen in lungs were scored based on the method developed by Madec and Kobisch (51). Tissue consolidation represented by darker patchy areas was more pronounced in sham-immunized animals (**Supplementary Figure S3**). Lesion scores were significantly lower in OV-cH6/1-cH8/1-immunized groups independent of the challenge virus (**Figures 8A, B**). Lesion scores were approximately 10-fold higher in the sham-immunized+Oh07 challenged animals in comparison to the vaccinated group. A similar trend was found for the Ca09 challenged swine, but the overall lesion score was lower. The differences in the severity of macroscopic lesions observed between Oh07 and Ca09 challenged groups were likely attributed to the increase in disease severity of the Oh07 strain compared to other contemporary strains in swine (52). Additionally, viral load in lung tissue lysates and BAL were determined at D42 (7 dpc) and expressed in genome copies mL^−1^ (log 10). Viral load in both lungs and BAL were 10-fold lower in vaccinated animals (**Figure 8B**). Clinical signs associated with vaccine-associated enhanced respiratory disease (VAERD) were not observed during this study. In summary, these results indicate that immunization with ORFV^Δ121^cH6/1-ORFV^Δ121^cH8/1-IAV-S was able to reduce viral load and lung lesions.

## 4 Discussion

The use of ORFV as a viral vector for vaccine delivery has proven efficient, inducing protective immune responses against numerous viral agents in swine (1–6). Certain genes of the virus encode viral IMPs, which have the ability to modulate the host’s innate and proinflammatory responses during infection (44). One sof these IMPs is *ORFV121*, which encodes for an inhibitor of the nuclear factor-kappa beta (NF-κB) signaling pathway, and functions as a virulence factor contributing to the pathogenicity of ORFV (7, 8). Given that the deletion of *ORFV121* leads to a marked attenuation of ORFV, this locus has been used by our group as an insertion site for heterologous viral genes in ORFV-based vectors (4, 16). The replacement of this gene by the chimeric HAs in the present study was based on previous studies demonstrating that *ORFV121* provides a suitable insertion site that enables the stable expression of heterologous genes and presents increased immunogenicity in livestock species (4, 16).

Another crucial aspect of designing poxvirus vector-based vaccine candidates is to elect an efficient promoter that expresses heterologous genes efficiently in the absence of active virus replication (53). This is particularly important due to the inefficient replication of this virus in swine cells, as evidenced by the marked growth defect observed in replication curves performed in primary STu cells here. Thus, it is desirable to ensure a high level of heterologous gene expression under limited or lack of virus replication in non-permissive cells by using strong early poxviral promoters (54). Recently, we conducted a transcriptomic analysis in ORFV-infected cells and identified an endogenous ORFV promoter (p116) that drives both early and late gene expression. This promoter not only resulted in higher early transgene expression, but also prolonged expression compared to well-known VACV promoters, such as I1L and vv7.5, probably as a result of a more efficient recognition by ORFV transcription machinery (46).

Several studies have emphasized the significance of conserved epitopes in driving broad cross-reactive humoral and cellular responses against IAV-S. Enhancement of HA stalk-specific antibody and memory B cells following sequential exposure to divergent viruses was demonstrated after the 2009 H1N1 pandemic in sera from infected individuals (55, 56). However, commercially available IAV-S vaccines primarily stimulate strain-specific antibodies that target the variable immunodominant globular head domain of HA (HA1), which is highly plastic and can lead to virus escape through antigenic drift (43, 57, 58). Consequently, the immunodominance of HA head weakens the antibody response against the stalk. In this study, we investigated a sequential immunization strategy using chimeric HAs delivered through an ORFV vector platform. Our results demonstrated that this approach raised cross-reactive antibody responses against divergent swine influenza viruses. This was likely achieved by inducing robust cross-reactive antibodies against conserved and subdominant epitopes present in the stalk region of HA, which ultimately provided protection as indicated by a reduced lung pathology and virus shedding post-challenge. Previously, similar findings were observed in mice and ferrets (38, 39, 42, 59, 60). Additional evidence from human trials suggests that HA stalk antibody levels increase in an age-dependent manner, with the elderly exhibiting the highest titers of antibodies against the H1 stalk, as well as the stalks of H3 and influenza B virus HAs, but at lower levels (61). Although the life span of stalk antibodies in circulation is controversial, a recent placebo-controlled phase I trial demonstrated that chimeric HA-based vaccines induce a strong long-lasting humoral response in healthy humans (43, 62). Indeed, a long-term investigation needs to be performed to assess the longevity of the antibodies, but overall, the available data support the potential of chimeric HA-based vaccines for protecting swine against IAV-S and warrant further exploration.

As anticipated, antibody levels elicited by ORFV-based platforms carrying the chimeric HAs seem lower when compared to those obtained with the ORFV strain expressing wild-type HA1 (ORFV-HA) (5). This can be attributed to the replacement of the most immunogenic domain head region with exotic heads, which may pose challenges for the antibodies to effectively interact with epitopes shielded within the stalk region. Similarly, the production of neutralizing antibodies against IAV-S may be reduced because they primarily target antigenic sites in the receptor binding site (RBS) in the HA head domain (63, 64). The role of these neutralizing antibodies is to block the virion binding to the receptor on the cell membrane, and consequently, viral entry into the host cell. In contrast to the minimal detection of neutralizing and hemagglutination inhibition (HI) antibody titers, high levels of total IgG, were detected in the serum of vaccinated swine. Although antibodies directed against the stalk region are not capable of preventing virus attachment, they can bind to HA2, and subsequently, prevent or affect the conformational changes triggered by the proteolytic cleavage of HA into HA1 and HA2. These conformational changes are required for membrane fusion, resulting in decreased entry and viral spread from infected to non-infected cells (65–69). Alternatively, antibodies targeting the stalk region can provide protection *in vivo* by engaging Fc-mediated effector functions, such as Ab-dependent cellular cytotoxicity (ADCC) and ADCP (Ab-dependent cell-mediated phagocytosis) (70–73). Although stalk-specific antibodies typically do not confer sterilizing protection, they are crucial in providing broad protection against lethal virus challenges (39, 73–76).

Since most antibodies against the stalk domain are non-neutralizing, they could potentially exacerbate disease presentation through activation of the antibody-dependent enhancement (ADE). This phenomenon is attributed to the binding of Fc region of sub-neutralizing and non-neutralizing antibodies to Fcγ receptor (FcγR) on the surface of immune cells, particularly on the phagocytic leucocyte subset (77, 78). Besides facilitating infection in non-permissive cell types, Fc-FcγR interactions have been suggested to play a detrimental role in the development of respiratory disease post-vaccination against viruses like influenza (79–81). This phenomenon termed vaccine-associated enhanced respiratory disease (VAERD) could preclude the use of the cHA platforms; however, in our study, we did not observe any clinical signs associated with VAERD following immunization, highlighting the safety of the ORFV^Δ121^cH6/1 and ORFV^Δ121^cH8/1 platforms in swine.

The findings of this study showed high levels of IgG in sera, and enhanced IgA antibody responses in BAL of immunized pigs that were reactive against homologous and heterologous swine influenza virus strains. Despite the anamnestic effect of the challenge virus on the level of IgA antibodies, vaccinated animals exhibited higher responses compared to the sham-immunized, suggesting that the IM prime–boost regimen with the vaccine candidates elicited cross-reactive immune responses at local mucosal sites. Existing secretory IgA (S-IgA) antibodies play an important role in rapidly neutralizing pathogens before they breach the mucosal barrier and enter the body (82). Moreover, inducing a strong mucosal immunity is particularly beneficial, as it has been linked to a broader spectrum of protection against influenza (82, 83).

Cellular-mediated responses are recognized for their critical role in the clearance of influenza virus infections, and they can act as a compensatory mechanism when the humoral response is weak, contributing to disease protection by reducing viral replication and shedding in the respiratory tract (84–88). While our study focused on the humoral response and cross-reactive antibodies against conserved epitopes of the stalk region, we acknowledge the importance of cellular-mediated responses in providing protection against divergent swine influenza viruses. In future studies, we aim to investigate the role of cross-reactive T cells targeting conserved epitopes and further explore the cellular aspect of the immune system in relation to this vaccine strategy.

Immunization with the cHA platforms in the present study resulted in cross-reactive antibody responses against broad divergent influenza A viruses. In animals challenged with the OH07 virus (gamma clade), we observed marked reductions in virus shedding in nasal secretions, viral load in lungs, and lung pathology. Although we did not observe a significant reduction in virus shedding in nasal secretions in animals challenged with CA09 (pndm clade) on days 1 and 3 pi, reduced viral RNA was detected on day 5 in nasal secretions, and reduced viral load in lungs and BAL as well as reduced lung lesions and pathology were observed in immunized animals challenged with CA09 when compared to the control animals. These observations confirm the potential of the cHA platforms in providing cross-reactive immunity and protection against IAV-S in swine.

The rational design of vaccines that target regions of the influenza virus with minimal mutation holds the potential to provide a wider breadth of protection against epidemics caused by emerging IAV-S. Furthermore, the delivery platform ORFV provides a promising avenue for the development of improved vaccine candidates aimed at effectively managing IAV-S infections in swine. While significant advancements have been made in the development of more efficient influenza vaccines, additional research is still needed to find a long-term solution for influenza. Inclusion of additional proteins in vector platforms like ORFV may be a good alternative to expand even further the immunogenicity and breadth of immune responses and protection elicited by this platform.

## Data availability statement

The data sequencing presented in the study are deposited in the Genbank and Bioproject repositories, under accession numbers PP211528, PP211529 and PRJNA1068754, respectively. Further inquiries can be directed to the corresponding author.

## Ethics statement

The animal study was conducted at Cornell University, adhering to the guidelines and protocols approved by the Institutional Animal Care and Use Committee (IACUC approval no. 2019-0041) and in accordance with the Animal Welfare Act Amendments. The study was conducted in accordance with the local legislation and institutional requirements.

## Author contributions

GM: Data curation, Formal analysis, Investigation, Methodology, Validation, Writing – original draft, Writing – review & editing. PS: Investigation, Methodology, Writing – review & editing. SB: Investigation, Methodology, Writing – review & editing, Data curation, Formal analysis. DD: Data curation, Formal analysis, Investigation, Methodology, Writing – review & editing, Conceptualization, Funding acquisition, Project administration, Resources, Supervision, Writing – original draft.

## Supplementary material

Supplementary Material 1 Chimeric HA sequences for cH1/6 and cH1/8 designed, synthesized and cloned into the ORFV vector in the present study.

Supplementary Figure 1 Experimental design of immunization-challenge study in pigs. Twenty-eight four-week old IAV-S seronegative piglets were allocated into four experimental groups as shown and subjected to prime-boost immunization regimen with OV-cH6/1- ORFV-cH8/1. Sham-immunized animals served as controls. On D35 animals were challenged with IAV-S H1N1 OH07 or CA09 as shown in the figure. Samples were collected as indicated to assess immune and virological responses.

Supplementary Figure 2 IAV-S strains used for the in-house whole-virus ELISA and their phylogenetically relationship with inclusion of the strain used as the stalk domain for the chimeric HAs.

Supplementary Figure 3 Macroscopic lesions found in lungs from **(A)** vaccinated OV-cH6/1- ORFV-cH8/1 and **(B)** sham-immunized piglets challenged with Oh07 virus strain, as well as **(C)** vaccinated OV-cH6/1- ORFV-cH8/1 and **(D)** sham-immunized piglets challenged with Ca09 virus strain. Signs of tissue consolidation can be identified by darker patchy areas, which are more noticeable in the sham-immunized animals.

## References

[1] Friebe A Siegling A Friederichs S Volk H-D Weber O. Immunomodulatory effects of inactivated parapoxvirus ovis (ORF virus) on human peripheral immune cells: induction of cytokine secretion in monocytes and Th1-like cells. J Virol (2004) 78:9400–11. doi: 10.1128/JVI.78.17.9400-9411.2004 PMC506965 15308734

[2] Voigt H Merant C Wienhold D Braun A Hutet E Le Potier MF. Efficient priming against classical swine fever with a safe glycoprotein E2 expressing Orf virus recombinant (ORFV VrV-E2). Vaccine (2007) 25:5915–26. doi: 10.1016/j.vaccine.2007.05.035 17600594

[3] Okda FA Lawson S Singrey A Nelson J Hain KS Joshi LR. The S2 glycoprotein subunit of porcine epidemic diarrhea virus contains immunodominant neutralizing epitopes. Virology (2017) 509:185–94. doi: 10.1016/j.virol.2017.06.013 PMC7111671 28647506

[4] Hain KS Joshi LR Okda F Nelson J Singrey A Lawson S. Immunogenicity of a recombinant parapoxvirus expressing the spike protein of porcine epidemic diarrhea virus. J Gen Virol (2016), 2719–31. doi: 10.1099/jgv.0.000586 27558814

[5] Joshi LR Knudsen D Piñeyro P Dhakal S Renukaradhya GJ Diel DG. Protective efficacy of an orf virus-vector encoding the hemagglutinin and the nucleoprotein of influenza A virus in swine. Front Immunol (2021) 12:747574. doi: 10.3389/fimmu.2021.747574 34804030 PMC8602839

[6] Joshi LR Okda FA Singrey A Maggioli MF Faccin TC Fernandes MHV. Passive immunity to porcine epidemic diarrhea virus following immunization of pregnant gilts with a recombinant orf virus vector expressing the spike protein. Arch Virol (2018) 163:2327–35. doi: 10.1007/s00705-018-3855-1 PMC7086649 29725899

[7] Diel DG Luo S Delhon G Peng Y Flores EF Rock DL. Orf virus *ORFV121* encodes a novel inhibitor of NF- B that contributes to virus virulence. J Virol (2011) 85:2037–49. doi: 10.1128/jvi.02236-10 PMC3067802 21177808

[8] Fleming SB McCaughan C Lateef Z Dunn A Wise LM Real NC. Deletion of the chemokine binding protein gene from the parapoxvirus ORF virus reduces virulence and pathogenesis in sheep. Front Microbiol (2017) 8:46. doi: 10.3389/fmicb.2017.00046 28174562 PMC5258736

[9] Fleming SB McCaughan CA Andrews AE Nash AD Mercer AA. A homolog of interleukin-10 is encoded by the poxvirus orf virus. J Virol (1997) 71:4857–61. doi: 10.1128/jvi.71.6.4857-4861.1997 PMC191714 9151886

[10] Deane D McInnes CJ Percival A Wood A Thomson J Lear A. Orf virus encodes a novel secreted protein inhibitor of granulocyte-macrophage colony-stimulating factor and interleukin-2. J Virol (2000) 74:1313–20. doi: 10.1128/JVI.74.3.1313-1320.2000 PMC111466 10627542

[11] Fischer T Planz O Stitz L Rziha H-J. Novel recombinant parapoxvirus vectors induce protective humoral and cellular immunity against lethal herpesvirus challenge infection in mice. J Virol (2003) 77:9312–23. doi: 10.1128/jvi.77.17.9312-9323.2003 PMC187421 12915547

[12] Reguzova A Ghosh M Müller M Rziha HJ Amann R. Orf virus-based vaccine vector D1701-V induces strong CD8+ T cell response against the transgene but not against ORFV-derived epitopes. Vaccines (2020) 8:1–17. doi: 10.3390/vaccines8020295 PMC7349966 32531997

[13] Haig DM Mercer AA. Ovine diseases. Orf. Vet Res (1998) 29:311–26. 9689744

[14] Büttner M Rziha HJ. Parapoxviruses: From the lesion to the viral genome. J Vet Med Ser B (2002) 49:7–16. doi: 10.1046/j.1439-0450.2002.00539.x 11911596

[15] Rohde J Schirrmeier H Granzow H Rziha H-J. A new recombinant Orf virus (ORFV, Parapoxvirus) protects rabbits against lethal infection with rabbit hemorrhagic disease virus (RHDV). Vaccine (2011) 29:9256–64. doi: 10.1016/j.vaccine.2011.09.121 22001119

[16] Martins M Joshi LR Rodrigues FS Anziliero D Frandoloso R Kutish GF. Immunogenicity of ORFV-based vectors expressing the rabies virus glycoprotein in livestock speciefile:///Users/gabrielamansano/Downloads/A New Rabies Vaccine Based on a Recombinant Orf Virus (Parapoxvirus) Expressing the Rabies Virus Gly.pdfs. Virology (2017) 511:229–39. doi: 10.1016/j.virol.2017.08.027 28898730

[17] Dory D Fischer T Béven V Cariolet R Rziha HJ Jestin A. Prime-boost immunization using DNA vaccine and recombinant Orf virus protects pigs against Pseudorabies virus (Herpes suid 1). Vaccine (2006) 24:6256–63. doi: 10.1016/j.vaccine.2006.05.078 16814432

[18] van Rooij EMA Rijsewijk FAM Moonen-Leusen HW Bianchi ATJ Rziha HJ. Comparison of different prime-boost regimes with DNA and recombinant Orf virus based vaccines expressing glycoprotein D of pseudorabies virus in pigs. Vaccine (2010) 28:1808–13. doi: 10.1016/j.vaccine.2009.12.004 20018271

[19] Amann R Rohde J Wulle U Conlee D Raue R Martinon O. A new rabies vaccine based on a recombinant orf virus (Parapoxvirus) expressing the rabies virus glycoprotein. J Virol (2012) 87:1618–30. doi: 10.1128/JVI.02470-12 PMC3554190 23175365

[20] Martins M Joshi LR Rodrigues FS Anziliero D Frandoloso R Kutish GF. Immunogenicity of ORFV-based vectors expressing the rabies virus glycoprotein in livestock species. Virology (2017) 511:229–39. doi: 10.1016/j.virol.2017.08.027 28898730

[21] Rohde J Amann R Rziha HJ. New Orf virus (Parapoxvirus) recombinant expressing H5 hemagglutinin protects mice against H5N1 and H1N1 influenza A virus. PloS One (2013) 8:e83802 . doi: 10.1371/journal.pone.0083802 24376753 PMC3869816

[22] Do Nascimento GM Bugybayeva D Patil V Schrock J Yadagiri G Renukaradhya GJ. An orf-virus (ORFV)-based vector expressing a consensus H1 hemagglutinin provides protection against diverse swine influenza viruses. Viruses (2023) 15:994. doi: 10.3390/V15040994 37112974 PMC10147081

[23] Newman AP Reisdorf E Beinemann J Uyeki TM Balish A Shu B. Human case of swine influenza A (H1N1) triple reassortant virus infection, Wisconsin. Emerg Infect Dis. (2006) 14(9):1470–2. doi: 10.3201/eid1409.080305 PMC2603093 18760023

[24] Skarlupka AL Owino SO Suzuki-Williams LP Crevar CJ Carter DM Ross TM. Computationally optimized broadly reactive vaccine based upon swine H1N1 influenza hemagglutinin sequences protects against both swine and human isolated viruses. Hum Vaccines Immunother (2019) 15:2013–29. doi: 10.1080/21645515.2019.1653743 PMC6773400 31448974

[25] Garten RJ Davis CT Russell CA Shu B Lindstrom S Balish A. Antigenic and genetic characteristics of swine-origin 2009 A(H1N1) influenza viruses circulating in humans. Sci (80- ) (2009) 325:197–201. doi: 10.1126/science.1176225 PMC3250984 19465683

[26] Palese P Schulman JL. Mapping of the influenza virus genome: Identification of the hemagglutinin and the neuraminidase genes. Proc Natl Acad Sci USA (1976) 73:2142–6. doi: 10.1073/pnas.73.6.2142 PMC430466 1064882

[27] Oxford JS Hockley DJ. “Orthomyxoviridae” Perspectives in Medical Virology (1987) 3:213–32.

[28] Thacker E Janke B. Swine influenza virus: zoonotic potential and vaccination strategies for the control of avian and swine influenzas. J Infect Dis (2008) 197:S19–24. doi: 10.1086/524988 18269323

[29] Bosch FX Orlich M Klenk HD Rott R. The structure of the hemagglutinin, a determinant for the pathogenicity of influenza viruses. Virology (1979) 95:197–207. doi: 10.1016/0042-6822(79)90414-8 442540

[30] Bullough PA Hughson FM Skehel JJ Wiley DC. Structure of influenza HA at the pH of membrane fusion. Nature (1994) 371:37–43. doi: 10.1038/371037a0 8072525

[31] Yassine HM Khatri M Zhang YJ Lee CW Byrum BA O’Quin J. Characterization of triple reassortant H1N1 influenza A viruses from swine in Ohio. Vet Microbiol (2009) 139:132–9. doi: 10.1016/j.vetmic.2009.04.028 19477087

[32] Kawaoka Y Webster RG. Sequence requirements for cleavage activation of influenza virus hemagglutinin expressed in mammalian cells. Proc Natl Acad Sci (1988) 85:324–8. doi: 10.1073/pnas.85.2.324 PMC279540 2829180

[33] Wang W DeFeo CJ Alvarado-Facundo E Vassell R Weiss CD. Intermonomer interactions in hemagglutinin subunits HA1 and HA2 affecting hemagglutinin stability and influenza virus infectivity. J Virol (2015) 89:10602–11. doi: 10.1128/jvi.00939-15 PMC4580181 26269180

[34] Skehel JJ Wiley DC. Receptor binding and membrane fusion in virus entry: the influenza hemagglutinin. Annu Rev Biochem (2000) 69:531–69. doi: 10.1146/annurev.biochem.69.1.531 10966468

[35] Benton DJ Nans A Calder LJ Turner J Neu U Lin YP. Influenza hemagglutinin membrane anchor. Proc Natl Acad Sci USA (2018) 115:10112–7. doi: 10.1073/pnas.1810927115 PMC6176637 30224494

[36] Krammer F Palese P. Universal influenza virus vaccines that target the conserved hemagglutinin stalk and conserved sites in the head domain. J Infect Dis (2019) 219:S62–7. doi: 10.1093/infdis/jiy711 PMC6452318 30715353

[37] Nachbagauer R Salaun B Stadlbauer D Behzadi MA Friel D Rajabhathor A. Pandemic influenza virus vaccines boost hemagglutinin stalk-specific antibody responses in primed adult and pediatric cohorts. NPJ Vaccines (2019) 4:1–12. doi: 10.1038/s41541-019-0147-z 31839997 PMC6898674

[38] Margine I Krammer F Hai R Heaton NS Tan GS Andrews SA. Hemagglutinin stalk-based universal vaccine constructs protect against group 2 influenza A viruses. J Virol (2013) 87:10435–46. doi: 10.1128/jvi.01715-13 PMC3807421 23903831

[39] Choi A Bouzya B Cortés Franco K-D Stadlbauer D Rajabhathor A Rouxel RN. Chimeric hemagglutinin-based influenza virus vaccines induce protective stalk-specific humoral immunity and cellular responses in mice. ImmunoHorizons (2019) 3:133–48. doi: 10.4049/immunohorizons.1900022 PMC6485968 31032479

[40] Nachbagauer R Miller MS Hai R Ryder AB Rose JK Palese P. Hemagglutinin stalk immunity reduces influenza virus replication and transmission in ferrets. J Virol (2016) 90:3268–73. doi: 10.1128/jvi.02481-15 PMC4810634 26719251

[41] Nachbagauer R Liu WC Choi A Wohlbold TJ Atlas T Rajendran M. A universal influenza virus vaccine candidate confers protection against pandemic H1N1 infection in preclinical ferret studies. NPJ Vaccines (2017) 2:1–12. doi: 10.1038/s41541-017-0026-4 29263881 PMC5627297

[42] Liu WC Nachbagauer R Stadlbauer D Strohmeier S Solórzano A Berlanda-Scorza F. Chimeric hemagglutinin-based live-attenuated vaccines confer durable protective immunity against influenza a viruses in a preclinical ferret model. Vaccines (2021) 9:1–18. doi: 10.3390/vaccines9010040 PMC7826668 33440898

[43] Nachbagauer R Feser J Naficy A Bernstein DI Guptill J Walter EB. A chimeric hemagglutinin-based universal influenza virus vaccine approach induces broad and long-lasting immunity in a randomized, placebo-controlled phase I trial. Nat Med (2021) 27:106–14. doi: 10.1038/s41591-020-1118-7 33288923

[44] Delhon G Tulman ER Afonso CL Lu Z de la Concha-Bermejillo A Lehmkuhl HD. Genomes of the parapoxviruses orf virus and bovine papular stomatitis virus. J Virol (2004) 78:168–77. doi: 10.1128/JVI.78.1.168-177.2004 PMC303426 14671098

[45] Diel DG Delhon G Luo S Flores EF Rock DL. A novel inhibitor of the NF-{kappa}B signaling pathway encoded by the parapoxvirus orf virus. J Virol (2010) 84:3962–73. doi: 10.1128/JVI.02291-09 PMC2849485 20147406

[46] Joshi LR do Nascimento GM Diel DG. The transcriptome of the parapoxvirus Orf virus reveals novel promoters for heterologous gene expression by poxvirus vectors. Virology (2023) 587:109864. doi: 10.1016/j.virol.2023.109864 37595395

[47] Liu X Kremer M Broyles SS. A natural vaccinia virus promoter with exceptional capacity to direct protein synthesis. J Virol Methods (2004), 141–5. doi: 10.1016/j.jviromet.2004.08.009 15542137

[48] Diel DG Luo S Delhon G Peng Y Flores EF Rock DL. A nuclear inhibitor of NF- B encoded by a poxvirus. J Virol (2011) 85:264–75. doi: 10.1128/jvi.01149-10 PMC3014193 20980501

[49] Do Nascimento GM Bugybayeva D Patil V Schrock J Yadagiri G Renukaradhya GJ. An orf-virus (ORFV)-based vector expressing a consensus H1 hemagglutinin provides protection against diverse swine influenza viruses. Viruses (2023) 15:994. doi: 10.3390/V15040994 37112974 PMC10147081

[50] Gauger PC Vincent AL. Enzyme-linked immunosorbent assay for detection of serum or mucosal isotype-specific igG and igA whole-virus antibody to influenza A virus in swine. Methods Mol Biol (2020) 2123:311–20. doi: 10.1007/978-1-0716-0346-8_22 32170697

[51] Madec F Kobisch M. Bilan lésionnel des poumons de porcs charcutiers à l’abattoir. Journées la Rech Porc (1982) 14:65–71. doi: 10.1016/0147-9571(85)90055-4

[52] Vincent AL Swenson SL Lager KM Gauger PC Loiacono C Zhang Y. Characterization of an influenza A virus isolated from pigs during an outbreak of respiratory disease in swine and people during a county fair in the United States. Vet Microbiol (2009) 137:51–9. doi: 10.1016/j.vetmic.2009.01.003 19203846

[53] Baldick CJ Moss B. Characterization and temporal regulation of mRNAs encoded by vaccinia virus intermediate-stage genes. J Virol (1993) 67:3515–27. doi: 10.1128/jvi.67.6.3515-3527.1993 PMC237698 8098779

[54] Alharbi NK. Poxviral promoters for improving the immunogenicity of MVA delivered vaccines. Hum Vaccines Immunother (2019) 15:203–9. doi: 10.1080/21645515.2018.1513439 PMC6363155 30148692

[55] Li GM Chiu C Wrammert J McCausland M Andrews SF Zheng NY. Pandemic H1N1 influenza vaccine induces a recall response in humans that favors broadly cross-reactive memory B cells. Proc Natl Acad Sci U.S.A. (2012) 109:9047–52. doi: 10.1073/pnas.1118979109 PMC3384143 22615367

[56] Wrammert J Koutsonanos D Li GM Edupuganti S Sui J Morrissey M. Broadly cross-reactive antibodies dominate the human B cell response against 2009 pandemic H1N1 influenza virus infection. J Exp Med (2011) 208:181–93. doi: 10.1084/jem.20101352 PMC3023136 21220454

[57] Doud MB Bloom JD. Accurate measurement of the effects of all amino-acid mutations on influenza hemagglutinin. Viruses (2016) 8:1–17. doi: 10.3390/v8060155 PMC4926175 27271655

[58] Kirkpatrick E Qiu X Wilson PC Bahl J Krammer F. The influenza virus hemagglutinin head evolves faster than the stalk domain. Sci Rep (2018) 8:1–14. doi: 10.1038/s41598-018-28706-1 29992986 PMC6041311

[59] Krammer F Pica N Hai R Margine I Palese P. Chimeric hemagglutinin influenza virus vaccine constructs elicit broadly protective stalk-specific antibodies. J Virol (2013) 87:6542–50. doi: 10.1128/JVI.00641-13 PMC3676110 23576508

[60] Nachbagauer R Kinzler D Choi A Hirsh A Beaulieu E Lecrenier N. A chimeric haemagglutinin-based influenza split virion vaccine adjuvanted with AS03 induces protective stalk-reactive antibodies in mice. NPJ Vaccines (2016) 1:1–10. doi: 10.1038/npjvaccines.2016.15 PMC5707880 29250436

[61] Nachbagauer R Choi A Izikson R Cox MM Palese P Krammer F. Age dependence and isotype specificity of influenza virus hemagglutinin stalk-reactive antibodies in humans. MBio (2016) 7:1–10. doi: 10.1128/mBio.01996-15 PMC4725014 26787832

[62] Bernstein DI Guptill J Naficy A Nachbagauer R Berlanda-Scorza F Feser J. Immunogenicity of chimeric haemagglutinin-based, universal influenza virus vaccine candidates: interim results of a randomised, placebo-controlled, phase 1 clinical trial. Lancet Infect Dis (2020) 20:80–91. doi: 10.1016/S1473-3099(19)30393-7 31630990 PMC6928577

[63] Caton AJ Brownlee GG Yewdell JW Gerhard W. The antigenic structure of the influenza virus A/PR/8/34 hemagglutinin (H1 subtype). Cell (1982) 31:417–27. doi: 10.1016/0092-8674(82)90135-0 6186384

[64] Wiley DC Wilson IA Skehel JJ. Structural identification of the antibody-binding sites of Hong Kong influenza haemagglutinin and their involvement in antigenic variation. Nature (1981) 289:373–8. doi: 10.1038/289373a0 6162101

[65] Sui J Hwang WC Perez S Wei G Aird D Chen LM. Structural and functional bases for broad-spectrum neutralization of avian and human influenza A viruses. Nat Struct Mol Biol (2009) 16:265–73. doi: 10.1038/nsmb.1566 PMC2692245 19234466

[66] Tan GS Krammer F Eggink D Kongchanagul A Moran TM Palese P. A pan-H1 anti-hemagglutinin monoclonal antibody with potent broad-spectrum efficacy *in vivo* . J Virol (2012) 86:6179–88. doi: 10.1128/jvi.00469-12 PMC3372189 22491456

[67] Dreyfus C Laursen NS Kwaks T Zuijdgeest D Khayat R Ekiert DC. Highly conserved protective epitopes on influenza B viruses. Science (2012) 337:1343–8. doi: 10.1126/science.1222908 PMC3538841 22878502

[68] Ekiert DC Friesen RHE Bhabha G Kwaks T Yu W Ophorst C. A highly conserved neutralizing epitope on group 2 influenza A viruses. Sci (80- ) (2011) 333:843–50. doi: 10.1126/science.1204839.A PMC3210727 21737702

[69] Ekiert DC Bhabha G Elsliger M Friesen RHE Jongeneelen M Throsby M. Antibody recognition of a highly conserved influenza virus epitope : implications for universal prevention and therapy. Sci (80- ) (2009) 324:246–51. doi: 10.1126/science.1171491.Antibody PMC2758658 19251591

[70] Dilillo DJ Tan GS Palese P Ravetch JV. Broadly neutralizing hemagglutinin stalk-specific antibodies require FcR interactions for protection against influenza virus. vivo. Nat Med (2014) 20:143–51. doi: 10.1038/nm.3443 PMC3966466 24412922

[71] DiLillo DJ Palese P Wilson PC Ravetch JV. Broadly neutralizing anti-influenza antibodies require Fc receptor engagement for in *vivo* protection. J Clin Invest (2016) 126:605–10. doi: 10.1172/JCI84428 PMC4731186 26731473

[72] Jegaskanda S Reading PC Kent SJ. Influenza-specific antibody-dependent cellular cytotoxicity: toward a universal influenza vaccine. J Immunol (2014) 193:469–75. doi: 10.4049/jimmunol.1400432 24994909

[73] He W Chen CJ Mullarkey CE Hamilton JR Wong CK Leon PE. Alveolar macrophages are critical for broadly-reactive antibody-mediated protection against influenza A virus in mice. Nat Commun (2017) 8:1–13. doi: 10.1038/s41467-017-00928-3 29018261 PMC5635038

[74] Guthmiller JJ Han J Utset HA Li L Lan LYL Henry C. Broadly neutralizing antibodies target a haemagglutinin anchor epitope. Nature (2022) 602:314–20. doi: 10.1038/s41586-021-04356-8 PMC8828479 34942633

[75] Paules CI Lakdawala S McAuliffe JM Paskel M Vogel L Kallewaard NL. The hemagglutinin A stem antibody MEDI8852 prevents and controls disease and limits transmission of pandemic influenza viruses. J Infect Dis (2017) 216:356–65. doi: 10.1093/infdis/jix292 PMC5853468 28633457

[76] Sutton TC Lamirande EW Bock KW Moore IN Koudstaal W Rehman M. *In vitro* neutralization is not predictive of prophylactic efficacy of broadly neutralizing monoclonal antibodies CR6261 and CR9114 against lethal H2 influenza virus challenge in mice. J Virol (2017) 91. doi: 10.1128/jvi.01603-17 PMC5709608 29046448

[77] Halstead SB O’Rourke EJ. Dengue viruses and mononuclear phagocytes. I. Infection enhancement by non-neutralizing antibody. J Exp Med (1977) 146:201–17. doi: 10.1084/jem.146.1.201 PMC2180729 406347

[78] Bournazos S Gupta A Ravetch JV. The role of IgG Fc receptors in antibody-dependent enhancement. Nat Rev Immunol (2020) 20:633–43. doi: 10.1038/s41577-020-00410-0 PMC7418887 32782358

[79] Khurana S Loving CL Manischewitz J King LR Gauger PC Henningson J. Vaccine-induced anti-HA2 antibodies promote virus fusion and enhance influenza virus respiratory disease. Sci Transl Med (2013) 5:200ra114. doi: 10.1126/scitranslmed.3006366 23986398

[80] Gauger PC Vincent AL Loving CL Henningson JN Lager KM Janke BH. Kinetics of lung lesion development and pro-inflammatory cytokine response in pigs with vaccine-associated enhanced respiratory disease induced by challenge with pandemic (2009) A/H1N1 influenza virus. Vet Pathol (2012) 49:900–12. doi: 10.1177/0300985812439724 22461226

[81] Vincent AL Lager KM Janke BH Gramer MR Richt JA. Failure of protection and enhanced pneumonia with a US H1N2 swine influenza virus in pigs vaccinated with an inactivated classical swine H1N1 vaccine. Vet Microbiol (2008) 126:310–23. doi: 10.1016/j.vetmic.2007.07.011 17719188

[82] van Riet E Ainai A Suzuki T Hasegawa H. Mucosal IgA responses in influenza virus infections; thoughts for vaccine design. Vaccine (2012) 30:5893–900. doi: 10.1016/j.vaccine.2012.04.109 22835738

[83] Dhakal S Renu S Ghimire S Lakshmanappa YS Hogshead BT Feliciano-Ruiz N. Mucosal immunity and protective efficacy of intranasal inactivated influenza vaccine is improved by chitosan nanoparticle delivery in pigs. Front Immunol (2018) 9:934. doi: 10.3389/fimmu.2018.00934 29770135 PMC5940749

[84] Zhong W Roberts AD Woodland DL. Antibody-independent antiviral function of memory CD4+ T cells in vivo requires regulatory signals from CD8+ effector T cells. J Immunol. (2001) 167(3):1379–86. doi: 10.4049/jimmunol.167.3.1379 11466356

[85] Wilkinson TM Li CKF Chui CSC Huang AKY Perkins M Liebner JC. Preexisting influenza-specific CD4 + T cells correlate with disease protection against influenza challenge in humans. Nat Med (2012) 18:274–80. doi: 10.1038/nm.2612 22286307

[86] Hogan RJ Zhong W Usherwood EJ Cookenham T Roberts AD Woodland DL. Protection from respiratory virus infections can be mediated by antigen-specific CD4+ T cells that persist in the lungs. J Exp Med (2001) 193:981–6. doi: 10.1084/jem.193.8.981 PMC2193400 11304559

[87] Hemann EA Kang S-M Legge KL. Protective CD8 T Cell–Mediated Immunity against Influenza A Virus Infection following Influenza Virus–like Particle Vaccination. J Immunol (2013) 191:2486–94. doi: 10.4049/jimmunol.1300954 PMC3777857 23885108

[88] Lu IN Farinelle S Sausy A Muller CP. Identification of a CD4 T-cell epitope in the hemagglutinin stalk domain of pandemic H1N1 influenza virus and its antigen-driven TCR usage signature in BALB/c mice. Cell Mol Immunol (2017) 14:511–20. doi: 10.1038/cmi.2016.20 PMC5518815 27157498

