# Supplementary material for: Immunogenicity of chimeric hemagglutinins delivered by an orf virus vector platform against swine influenza virus
Source: Front Immunol. 2024 Feb 28;15:1322879. doi: 10.3389/fimmu.2024.1322879 (PMC10933025; doi:10.3389/fimmu.2024.1322879)
Supplement: Supplementary Material 1 — Chimeric HA sequences for cH1/6 and cH1/8 designed, synthesized and cloned into the ORFV vector in the present study. [file DataSheet_1.docx]

Supplementary Material 1

Nucleotide and amino acid sequences of chimeric HAs: cH6/1 and cH8/1.

1. cH6/1-Flag tag

ATGATTGCAATCATTGTAATAGCGATATTGGCAACAGCCGGAAGATCAGACAAGATCTGCATTGGATATCATGCCAACAATTCAACAACGCAAGTGGATACTATACTTGAGAAAAATGTCACCGTCACACACTCAGTTGAACTGCTAGAGAACCAGAAGGAAGAAAGATTCTGCAAGATCTTAAACAAGGCCCCCCTCGACCTAAGAGGATGCACCATAGAAGGTTGGATCCTGGGGAATCCTCAATGCGACCTATTGCTTGGTGATCAAAGCTGGTCATATATAGTAGAAAGGCCTACTGCTCAAAATGGGATTTGCTACCCAGGAGCTTTGAGCGAAGTAGAAGAGTTGAAGGCACTTATTGGATCAGGAGAAAGGGTAGAGAGATTTGAGATGTTTCCCAAAAGTACATGGACAGGAGTAGACACCAGCAGTGGGGTAACAAGGGCTTGCCCTTATAATAGTGGTTCATCTTTCTATAGAAACCTCTTATGGATAATAAAGACCAAGTCAGCAGCATATCCAGTAATTAAAGGAACTTACAACAATACTGGAAATCAGCCAATCCTTTATTTCTGGGGTGTGCACCATCCACCTGACACCAATGAGCAAAATACCCTATATGGCTCTGGTGATAGGTACGTTAGAATGGGGACTGAAAGCATGAATTTTGCCAAGAGTCCGGAAATTGCTGCAAGACCTGCTGTGAAAGGTCAAAGAGGCAGAATTGACTATTACTGGTCTGTTTTAAAACCAGGAGAGACCTTGAATGTCGAATCCAATGGAAATCTAATTGCCCCTTGGTATGCATACAAATTTGTCAGCACAAACAATAAAGGAGCCGTCTTCAAGTCAAATTTACCAATCGAGAACTGTGATGCCACATGCCAGACTATTGCAGGAGTCTTAAGGACCAATAAAACATTTCAGAATGTGAGTCCTCTGTGGATAGGAGAATGCCCCAAATATGTAAAAAGTGAAAGTTTGAGGCTTGCAACTGGACTGAGAAATGTTCCACAGATTGAGACTAGAGGCCTATTCGGGGCCATTGCTGGCTTCATCGAAGGGGGGTGGACAGGGATGGTAGATGGATGGTACGGTTATCACCATCAAAATGAGCAGGGGTCAGGATATGCAGCCGATCTGAAGAGCACACAAAATGCCATTGATAAGATTACTAACAAAGTAAATTCTGTTATTGAAAAGATGAATACACAGTTCACAGCAGTTGGTAAAGAGTTCAACCACCTTGAAAAAAGAATAGAGAATCTAAATAAAAAGGTTGATGATGGTTTCCTGGACATTTGGACTTACAATGCCGAACTGTTGGTTCTACTGGAAAACGAAAGAACTTTGGACTATCACGATTCAAATGTGAAGAACTTGTATGAAAAAGTAAGAAACCAGTTAAAAAACAATGCCAAGGAAATTGGAAACGGCTGCTTTGAATTTTACCACAAATGCGACAACACATGCATGGAAAGTGTCAAGAATGGGACTTATGACTACCCAAAATACTCAGAGGAAGCAAAATTAAACAGAGAAAAAATAGATGGAGTAAAGCTGGACTCAACAAAGATCTACCAGATTTTGGCGATCTATTCAACTGTTGCCAGTTCATTGGTACTGGTAGTCTCCCTGGGGGCAATCAGCTTCTGGATGTGCTCTAATGGGTCTCTACAGTGTAGAATATGTATTGACTACAAAGACGATGACGACAAGTAA

Translation:

MIAIIVIAILATAGRSDKICIGYHANNSTTQVDTILEKNVTVTHSVELLENQKEERFCKILNKAPLDLRGCTIEGWILGNPQCDLLLGDQSWSYIVERPTAQNGICYPGALSEVEELKALIGSGERVERFEMFPKSTWTGVDTSSGVTRACPYNSGSSFYRNLLWIIKTKSAAYPVIKGTYNNTGNQPILYFWGVHHPPDTNEQNTLYGSGDRYVRMGTESMNFAKSPEIAARPAVKGQRGRIDYYWSVLKPGETLNVESNGNLIAPWYAYKFVSTNNKGAVFKSNLPIENCDATCQTIAGVLRTNKTFQNVSPLWIGECPKYVKSESLRLATGLRNVPQIETRGLFGAIAGFIEGGWTGMVDGWYGYHHQNEQGSGYAADLKSTQNAIDKITNKVNSVIEKMNTQFTAVGKEFNHLEKRIENLNKKVDDGFLDIWTYNAELLVLLENERTLDYHDSNVKNLYEKVRNQLKNNAKEIGNGCFEFYHKCDNTCMESVKNGTYDYPKYSEEAKLNREKIDGVKLDSTKIYQILAIYSTVASSLVLVVSLGAISFWMCSNGSLQCRICIDYKDDDDK

1. cH8/1-Flag tag

ATGGAGAAGTTTATCGCAATAGCAATGCTCTTGGCGAGCACAAATGCATACGATAGGATATGCATTGGTTACCAGTCGAACAACTCCACAGACACGGTGAACACTCTTATAGAGCAGAATGTACCAGTCACTCAAACAATGGAGCTTGTGGAAACAGAGAAACATCCCGCTTATTGTAACACTGATTTAGGAACGCCATTGGAACTGCGAGACTGCAAAATTGAGGCGGTAATATATGGGAATCCCAAGTGTGACATTCACCTAAAGGATCAAGGTTGGTCATACATAGTGGAGAGGCCCAGTGCGCCAGAGGGAATGTGTTATCCTGGATCAGTAGAAAATCTAGAGGAACTGAGATTTGTCTTTTCCAACGCGGCGTCCTATAAGAGGATAAGACTATTTGACTATTCCAGGTGGAATGTAACCAGCTCTGGGACCAGCAAGGCATGCAATGCATCAACAGGTGGTCAATCCTTTTATAGAAGCATCAATTGGTTGACCAAAAAGAAACCAGACACTTATGATTTCAATGAGGGAAGCTATATCAACAACGAAGATGGGGACATCATTTTCCTATGGGGGATCCATCATCCGCCTAACACAAAAGAGCAGACAACGCTGTACAAGAATGCAAACACTTTGAGTAGTGTTACTACCAACACCATAAACAGAAGCTTTCAACCCAATATCGGCCCAAGACCATTAGTCAGAGGACAACAAGGAAGAATGGATTACTATTGGGGCATCCTGAAAAGAGGAGAGACTCTGAAGATCAGGACCAATGGAAACTTAATTGCACCTGAATTTGGATATCTATTTAAGGGTGAAAGCCATGGCAGAATAATTCAAAATGAGGACATACCCATTGGAAACTGTCACACAAAATGCCAGACATATGCAGGAGCAATCAATAGCAGCAAACCCTTTCAAAATGCAAGTAGACATTATATGGGGGAATGTCCCAAATATGTGAAAAAGGCAAGCTTACGGCTTGCAGTGGGTCTTAGAAATACACCTTCTATTGAGCCCAAAGGCCTATTCGGGGCCATTGCTGGCTTCATCGAAGGGGGGTGGACAGGGATGGTAGATGGATGGTACGGTTATCACCATCAAAATGAGCAGGGGTCAGGATATGCAGCCGATCTGAAGAGCACACAAAATGCCATTGATAAGATTACTAACAAAGTAAATTCTGTTATTGAAAAGATGAATACACAGTTCACAGCAGTTGGTAAAGAGTTCAACCACCTTGAAAAAAGAATAGAGAATCTAAATAAAAAGGTTGATGATGGTTTCCTGGACATTTGGACTTACAATGCCGAACTGTTGGTTCTACTGGAAAACGAAAGAACTTTGGACTATCACGATTCAAATGTGAAGAACTTGTATGAAAAAGTAAGAAACCAGTTAAAAAACAATGCCAAGGAAATTGGAAACGGCTGCTTTGAATTTTACCACAAATGCGACAACACATGCATGGAAAGTGTCAAGAATGGGACTTATGACTACCCAAAATACTCAGAGGAAGCAAAATTAAACAGAGAAAAAATAGATGGAGTAAAGCTGGACTCAACAAAGATCTACCAGATTTTGGCGATCTATTCAACTGTTGCCAGTTCATTGGTACTGGTAGTCTCCCTGGGGGCAATCAGCTTCTGGATGTGCTCTAATGGGTCTCTACAGTGTAGAATATGTATTGACTACAAAGACGATGACGACAAGTAA

Translation:

MEKFIAIAMLLASTNAYDRICIGYQSNNSTDTVNTLIEQNVPVTQTMELVETEKHPAYCNTDLGTPLELRDCKIEAVIYGNPKCDIHLKDQGWSYIVERPSAPEGMCYPGSVENLEELRFVFSNAASYKRIRLFDYSRWNVTSSGTSKACNASTGGQSFYRSINWLTKKKPDTYDFNEGSYINNEDGDIIFLWGIHHPPNTKEQTTLYKNANTLSSVTTNTINRSFQPNIGPRPLVRGQQGRMDYYWGILKRGETLKIRTNGNLIAPEFGYLFKGESHGRIIQNEDIPIGNCHTKCQTYAGAINSSKPFQNASRHYMGECPKYVKKASLRLAVGLRNTPSIEPKGLFGAIAGFIEGGWTGMVDGWYGYHHQNEQGSGYAADLKSTQNAIDKITNKVNSVIEKMNTQFTAVGKEFNHLEKRIENLNKKVDDGFLDIWTYNAELLVLLENERTLDYHDSNVKNLYEKVRNQLKNNAKEIGNGCFEFYHKCDNTCMESVKNGTYDYPKYSEEAKLNREKIDGVKLDSTKIYQILAIYSTVASSLVLVVSLGAISFWMCSNGSLQCRICIDYKDDDDK
