# Supplementary figures and images for: Immunogenicity of chimeric hemagglutinins delivered by an orf virus vector platform against swine influenza virus
Source: Front Immunol. 2024 Feb 28;15:1322879. doi: 10.3389/fimmu.2024.1322879 (PMC10933025; doi:10.3389/fimmu.2024.1322879)

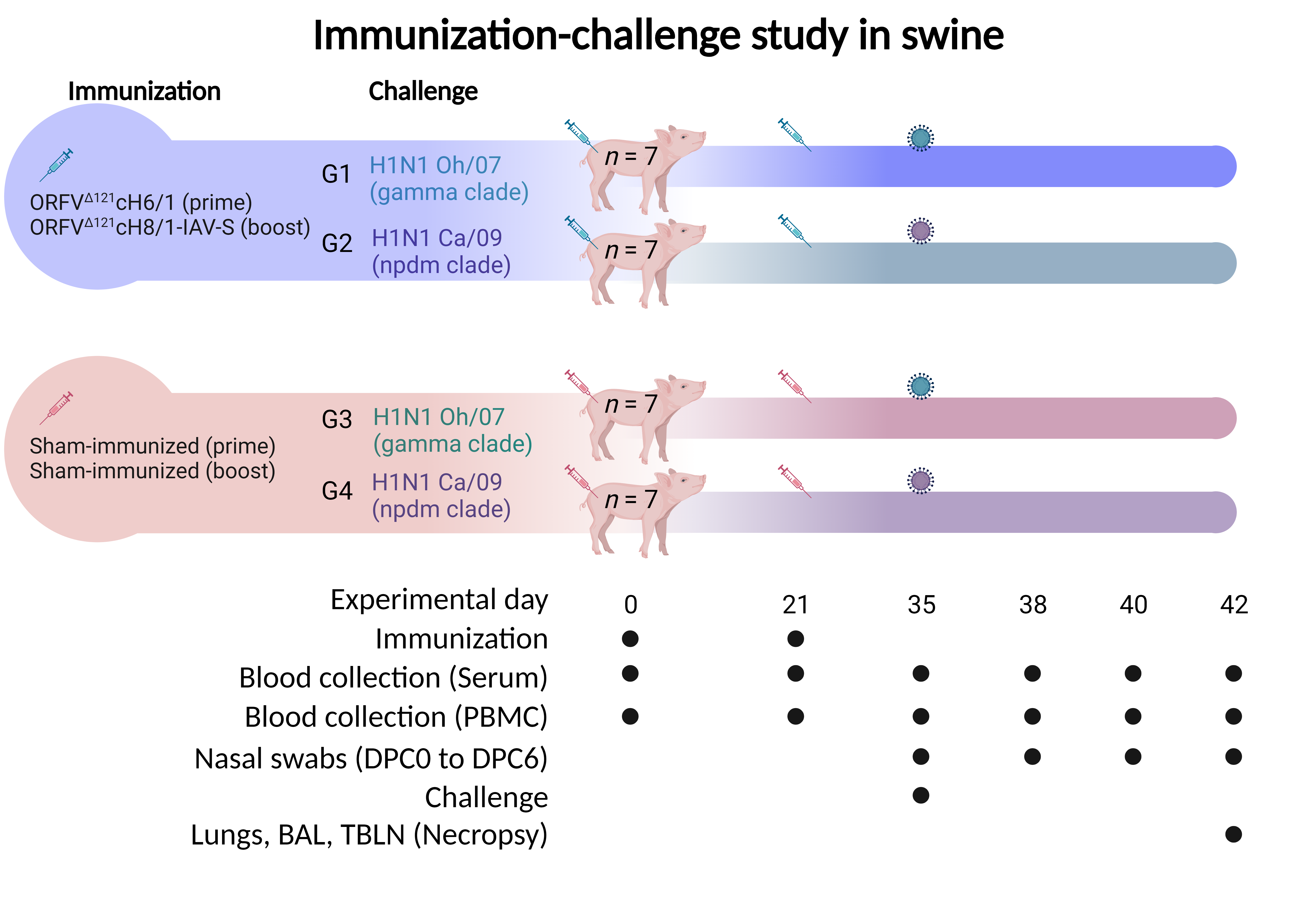

Supplement: Supplementary Figure 1 — Experimental design of immunization-challenge study in pigs. Twenty-eight four-week old IAV-S seronegative piglets were allocated into four experimental groups as shown and subjected to prime-boost immunization regimen with OV-cH6/1- ORFV-cH8/1. Sham-immunized animals served as controls. On D35 animals were challenged with IAV-S H1N1 OH07 or CA09 as shown in the figure. Samples were collected as indicated to assess immune and virological responses. [file Image_1.png]

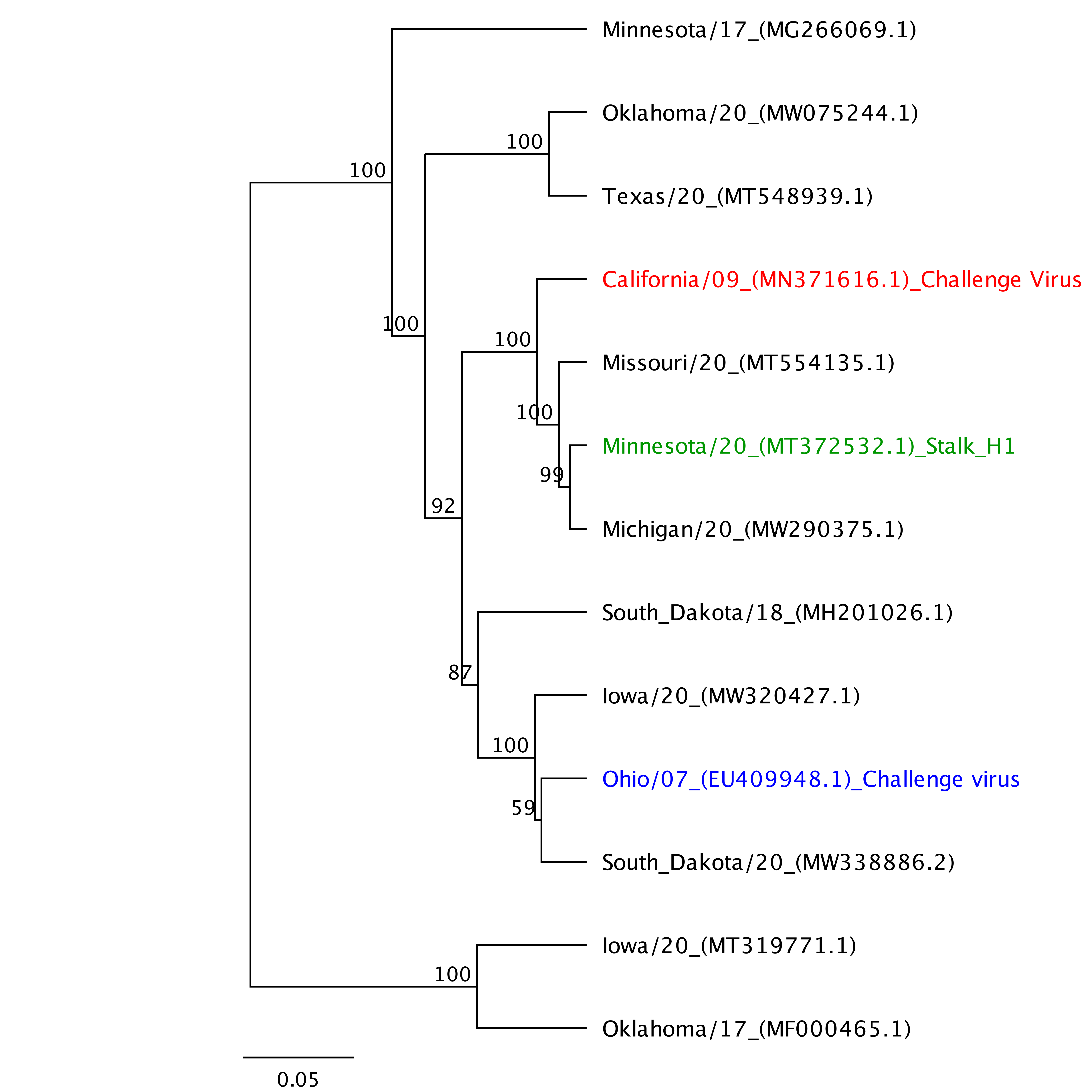

Supplement: Supplementary Figure 2 — IAV-S strains used for the in-house whole-virus ELISA and their phylogenetically relationship with inclusion of the strain used as the stalk domain for the chimeric HAs. [file Image_2.png]

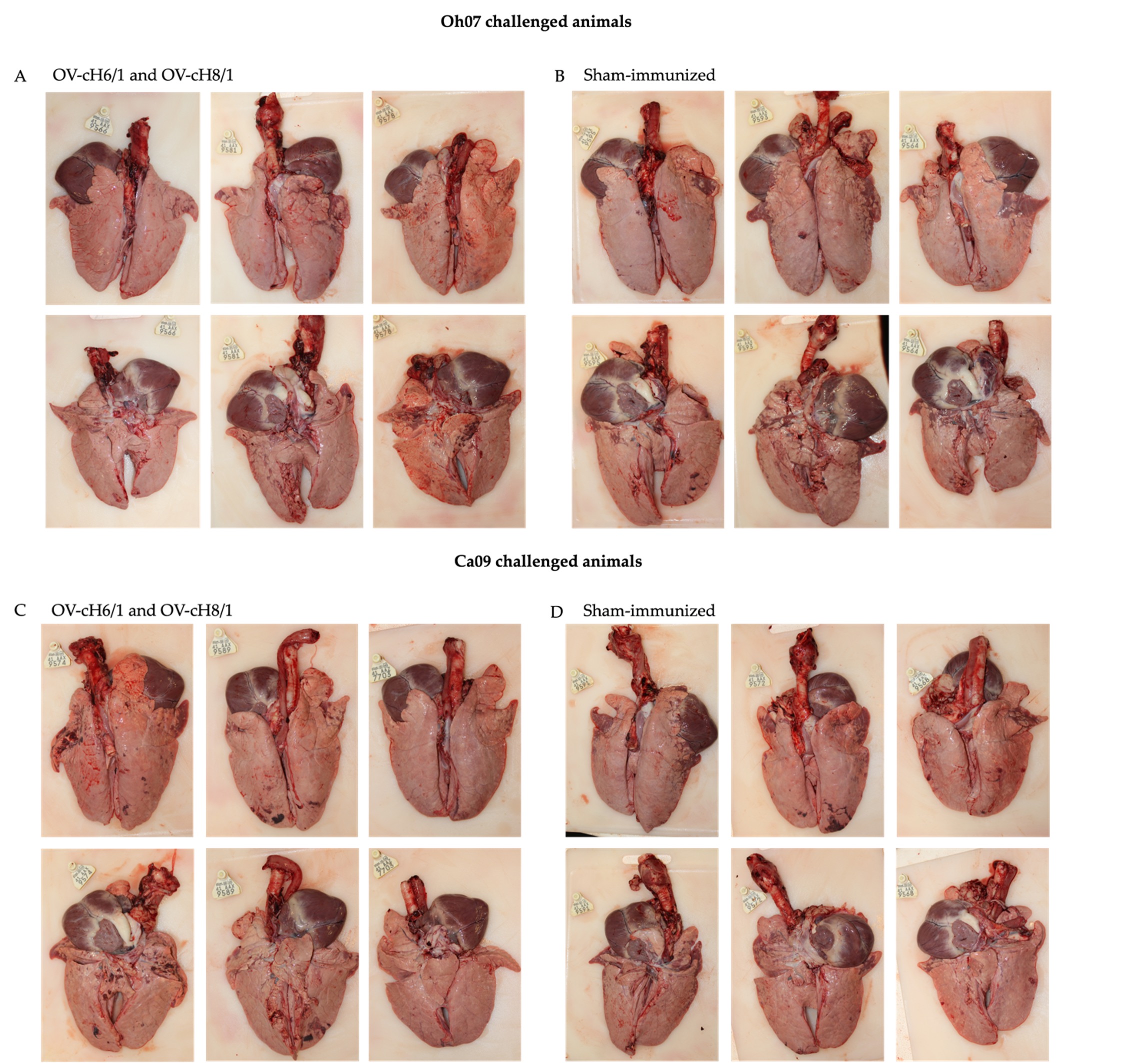

Supplement: Supplementary Figure 3 — Macroscopic lesions found in lungs from (A) vaccinated OV-cH6/1- ORFV-cH8/1 and (B) sham-immunized piglets challenged with Oh07 virus strain, as well as (C) vaccinated OV-cH6/1- ORFV-cH8/1 and (D) sham-immunized piglets challenged with Ca09 virus strain. Signs of tissue consolidation can be identified by darker patchy areas, which are more noticeable in the sham-immunized animals. [file Image_3.jpeg]
